# Magnetic resonance imaging features differentiate histologic and molecular subtypes of glioblastoma IDH-Wild type CNS WHO grade 4

**DOI:** 10.1007/s11060-026-05431-8

**Published:** 2026-01-21

**Authors:** Sohil H. Patel, Shanna Mayorov, Wooil Kim, Kanwar Singh, James R. Loftus, James T. Patrie, Prem P. Batchala, Allen Ko, Matthew D. Lee, Rajan Jain, David Schiff

**Affiliations:** 1 Department of Radiology and Imaging Sciences, Indiana University Health, 550 N. University Blvd. Indianapolis, Indianapolis, IN 46202 USA; 2 Department of Radiology and Medical Imaging, University of Virginia Health System, Charlottesville, VA USA; 3 Department of Radiology, New York University School of Medicine, 550 1st Avenue, New York, NY 10016 USA; 4 Department of Public Health Sciences, University of Virginia Health System, Charlottesville, VA USA; 5 Department of Neurosurgery, New York University School of Medicine, 550 1st Avenue, New York, NY 10016 USA; 6 Division of Neuro-Oncology, University of Virginia Health System, Charlottesville, VA USA

**Keywords:** MRI, Glioma, Glioblastoma, Isocitrate Dehydrogenase (IDH), World Health Organization (WHO)

## Abstract

**Purpose:**

Glioblastoma IDH-wild type, CNS WHO grade 4 (GBM) can be diagnosed on the basis of histologic features (histological-GBM) or molecular features (molecular-GBM). Only few studies report neuroimaging features of GBM in its modern classification, and none have controlled for surgical status or used multiple logistic regression analysis to determine unique predictors. Our study aimed to validate MRI features that distinguish histological-GBM and molecular-GBM.

**Methods:**

We analyzed a training cohort (*n* = 255) and validation cohort (*n* = 44) of GBM cases, classified according to the 2021 WHO Classification of Tumors of the CNS. For the training cohort, univariate and multiple logistic regression analyses determined if MRI metrics (contrast enhancement, ring-enhancement, vasogenic edema, multifocal tumor, lesion diameter, hemorrhage, number of lobes, and normalized ADC) and surgery type (biopsy vs. resection) predicted GBM-type (histological vs. molecular). A reduced multiple logistic regression model was constructed and applied to the validation dataset.

**Results:**

There were 231 histological-GBMs and 24 molecular-GBMs in the training cohort. Multiple logistic regression analysis including both MRI metrics and surgery type showed that contrast enhancement (OR 7.83 [95%CI: 1.23–49.68], *p* = 0.029), ring enhancement (OR 5.98 [95%CI: 1.09–32.93, *p* = 0.040), and normalized ADC (OR 0.78 [95%CI: 0.62–0.99], *p* = 0.039) differed between histological and molecular-GBM. Analysis of the validation dataset using the unique training dataset-derived predictor variables (contrast-enhancement, ring-enhancement, and normalized ADC) found correct classification of each histological and molecular-GBM.

**Conclusion:**

Molecular and histological-GBM exhibit distinct MRI phenotypes independent of surgical status.

**Supplementary Information:**

The online version contains supplementary material available at 10.1007/s11060-026-05431-8.

## Introduction

Since the 2016 World Health Organization (WHO) classification of CNS tumors, it was recognized that the majority of “lower grade” (i.e. WHO grade II and III) IDH-wild type (IDH-wt) diffuse astrocytomas behave in a similar manner to Glioblastoma (GBM) IDH-wt WHO grade IV. Although such tumors lacked necrosis or microvascular proliferation, they harbored molecular features consistent with GBM, including TERT promotor mutation, EGFR amplification, and/or a combined chromosome 7 gain and chromosome 10 loss (+ 7/-10) [1, 2]. As such, in the 2021 (and current) WHO classification of CNS tumors, the diagnosis “Glioblastoma, IDH-wild type, CNS-WHO grade 4” includes diffusely infiltrative astrocytic neoplasms lacking an IDH mutation that have either (1) histologic evidence of necrosis or microvascular proliferation, or (2) any of the following molecular alterations: TERT promotor mutation, EGFR amplification, or combined chromosome + 7/-10 [3].

The neuroimaging correlates of histologically confirmed GBM have been well characterized [4]. However, only few studies have reported GBM neuroimaging features in the context of the updated WHO 2021 classification, where the diagnosis of GBM IDH-wt can be determined with histological criteria (histological-GBM) or molecular criteria (molecular-GBM) [5–9]. The available literature indicates that most molecular-GBMs have minimal or no contrast-enhancement [5–8]. This may pose a diagnostic challenge in the pre-operative setting where they may be mistaken for lower grade neoplasms such as IDH-mutant gliomas, which frequently also lack contrast enhancement. Preliminary data suggests that advanced MRI metrics (diffusion and perfusion weighted MRI) might also help to distinguish molecular-GBM from IDH-mutant gliomas [10]. The biological basis for the differing MRI features of molecular and histological-GBM remains to be understood. Among comparable studies investigating molecular vs. histological-GBM, none have assessed differences in MRI features of hemorrhage and ADC and none have accounted for the potential confounder of surgery type (biopsy versus resection). In this study, we attempt to address this knowledge gap by analyzing a training cohort and validation cohort of GBM IDH-wt classified according to the 2021 WHO scheme. Our aim was to determine whether MRI features and/or surgery status differed between histological-GBM and molecular-GBM.

## Methods

This retrospective study evaluated MRI metrics and clinical information associated with histological-GBM vs. molecular-GBM using training and validation cohorts from our institutions. Institutional Review Board (IRB) approval was obtained for this HIPAA-compliant study.

### Patient selection

The training cohort was selected from a neuropathology database at University of Virginia Health containing 262 consecutive GBM IDH-wt cases diagnosed between 2018 and 2023. Inclusion criteria consisted of (1) a pathologic diagnosis of GBM IDH-wt rendered according to the diagnostic criteria of the WHO 2021 classification system; (2) pre-operative MRI with the minimum following pulse sequences: T2*WI/SWI, T2WI/FLAIR, DWI, pre-contrast T1WI, post-contrast T1WI. A total of 7 cases from the training cohort institution were excluded due to lack of minimum required pre-operative MRI data. Of the 255 GBM IDH-wt cases in the training cohort, there were 231 histological-GBMs and 24 molecular-GBMs. A validation cohort of 44 GBM cases (12 molecular-GBMs, 32 age-matched histological-GBMs) was selected from a GBM registry at New York University Medical Center. Demographic data, surgical status (biopsy versus resection), histopathology, and molecular data were all obtained from the electronic medical record. Relevant histological and molecular testing (IDH mutation, TERT promoter mutation, EGFR amplification, Chromosome + 7/-10) was performed according to standard clinical protocol and meeting the WHO 2021 criteria in our Clinical Laboratory Improvement Amendments certified neuropathology laboratories. Histological-GBMs in our cohorts had histological evidence of microvascular proliferation and/or necrosis. Molecular-GBMs in our cohorts lacked histological evidence of microvascular proliferation or necrosis, but had at least one of the following molecular markers: TERT promoter mutation, EGFR amplification, Chromosome + 7/-10. MRI data was acquired on both 1.5T and 3T scanners; for reference, we list the pre-operative MRI parameters for the training institution in Supplemental Table S1.

### Neuroimaging analysis

For the training cohort, pre-operative MRI scans were analyzed in consensus by two board-certified neuroradiologists with 4 and 12 years of experience, respectively, in a blinded fashion. Reproducible-neuroimaging metrics were adopted from prior publications [11, 12], and determined in consensus by the neuroradiologist readers. For each case, readers determined (1) presence/absence of contrast enhancement; (2) presence/absence of ring enhancement; (3) presence/absence of vasogenic edema (per Lasocki et al. [13]); (4) presence/absence of multifocal disease; (5) size of whole tumor (long axis diameter, cm); (6) presence/absence of hemorrhage; 8) number of lobes involved; 9) normalized ADC (minimum lesional ADC divided by ADC of normal appearing contralateral white matter, excluding hemorrhage).

Subsequently, an independent board-certified neuroradiologist with 2 years of experience analyzed the MRI scans of the validation cohort in a blinded fashion, recording those MRI metrics that were unique predictors of GBM-type based on the multivariate analysis of the training dataset.

### Statistical analysis

#### Training dataset analysis

In the training dataset analysis, univariate and multiple logistic regression was conducted to assess unadjusted and adjusted associations, respectively, between the a-priori selected set of MRI predictors/surgical status and GBM type (histological-GBM, molecular-GBM). Univariate bivariate associations and multivariate adjusted bivariate associations were identified via Wald chi-square tests and multiple logistic regression multicollinearity was examined by the variance inflation factor (VIF) to determine the degree to which regression coefficients standard errors were inflated by due to interdependencies between model predictors.

A reduced multiple logistic regression model was then constructed in which the predictors of GBM type were the set of multiple logistic regression predictors identified in step 1 as uniquely associated with GBM type at the 0.05 significance level. Based on the reduced multiple logistic regression model predicted probabilities for histological-GBM, a receiver operating characteristic (ROC) analysis was conducted to identify the optimum predicted probability cut-point (p*) for correctly classifying the training dataset patients as either a histological-GBM or a molecular-GBM. The optimum classification predicted probability cut-point (p*) was identified via the Youden J statistic, where the Youden J statistic = *classification sensitivity* + *classification specificity* – 1 [14].

#### Validation dataset analyses

Utilizing the training set reduced multiple logistic regression model regression coefficients, predicted probabilities for the histological-GBM were obtained for the validation dataset patients by inserting validation dataset values of the predictors of the reduced multiple logistic regression model into the reduced multiple logistic regression equation and converting the resulting predicted log-odds values (i.e., ln(θ)) to the probability (p) scale (i.e., p = e^ln(θ)^/1 + e^ln(θ^). Based on the validation dataset patients predicted probabilities for histological-GBM, patients were classified as either histological-GBM or molecular-GBM based on whether the patients predicted probability was less than, or greater or equal to p* established in the training dataset analysis. Diagnostic performance was assessed via sensitivity, specificity, positive predictive value (PPV), negative predictive value (NPV), false positive error rate (FPER), false negative error rate (FNER) and accuracy (A). Confidence interval construction for the diagnostic performance measures was based on the binomial-exact method of Agresti et al. [15].

## Results

### Training dataset analysis

**Fig. 1:**
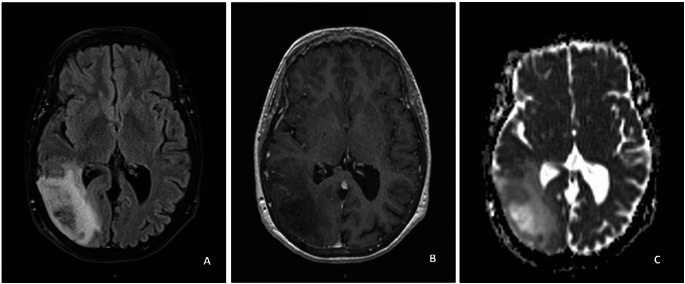
Molecular GBM. 64 year-old female with right temporal-occipital Glioblastoma, IDH-wild type, CNS-WHO grade 4. Histologically, there was no evidence of microvascular proliferation or necrosis. Molecular testing revealed a TERT-promoter C228T mutation and chromosome 7 gain/10 loss. (**A**) FLAIR sequence shows a predominantly hyperintense infiltrative mass. (**B**) Contrast-enhanced T1WI shows no contrast enhancement of the mass. (**C**) ADC map shows predominantly high signal. Minimum normalized ADC was 1.6

**Fig. 2:**
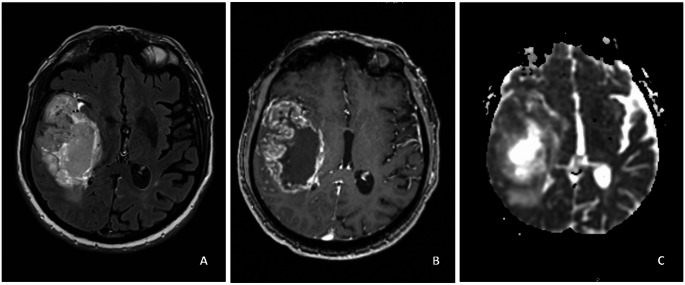
Histological GBM. 70 year old male with right insular-temporal Glioblastoma, IDH-wild type, CNS WHO grade 4. Histological evidence of necrosis and microvascular proliferation was present. (**A**) FLAIR sequence shows a heterogenous hyperintense mass. (**B**) Contrast-enhanced T1WI shows marked, thick ring-enhancement surrounding a central necrotic cavity. (**C**) ADC map shows regions of low signal corresponding to the contrast-enhancing tumor. Minimum normalized ADC was 0.9

Among the 255 GBM cases in the training dataset, there were 231 histological-GBMs and 24 molecular-GBMs. There were 108 females (42.4%) and 147 males (57.6%). Median age was 65.0 years (interquartile range [IQR]: 58.0–72.0). There was no significant difference in age or gender between molecular-GBM and histological-GBM. Among the histological-GBMs, necrosis was found in 90% of pathological samples, and microvascular proliferation was found in 97.8% of pathological samples. Among the molecular-GBMs, 21 were found to have a TERT promoter mutation, 5 were found to have EGFR amplification, and 7 were found to have chromosome + 7/-10. There were 7 molecular-GBMs that were discovered to have more than one of these molecular alterations. 163 patients underwent surgical resection and 92 patients underwent biopsy. Figures 1 and 2 are representative cases of molecular and histological-GBM, respectively.

Tabulated data and univariate logistic regression associations between GBM type and MRI metrics and surgery type are displayed in Table 1. Histological-GBM was positively associated with the presence of contrast enhancement, ring enhancement, vasogenic edema, and hemorrhage as well as lower normalized ADC (*p* < 0.001, all). Surgical biopsy was associated with molecular-GBM (*p* < 0.001).

**Table 1:** Tabulated data and univariate associations between MRI metrics/surgical status and GBM type in the training cohort

Predictor variable	Histological GBM	Molecular GBM	*P*-value
Contrast enhancement	Present: 227 (93.8%) Absent: 4 (1.7%)	Present: 9 (37.5%) Absent: 15 (62.5%)	**< 0.001**
Ring enhancement	Present: 188 (81.4%) Absent: 43 (18.6%)	Present: 3 (12.5%) Absent: 21 (87.5%)	**< 0.001**
Vasogenic edema	Present: 227 (98.3%) Absent: 4 (1.7%)	Present: 19 (79.2%) Absent: 5 (20.8%)	**< 0.001**
Multifocal	Present: 46 (19.9%) Absent: 185 (80.1%)	Present: 6 (25.0%) Absent: 18 (75.0%)	0.557
Maximum lesional diameter (cm)*	7.3 [5.3, 9.2]	6.35 [5.1, 7.3]	0.127
Hemorrhage	Present: 195 (84.4%) Absent: 36 (15.6%)	Present: 6 (25.0%) Absent: 18 (75.0%)	**< 0.001**
Number of lobes involved*	2 [2, 3]	2.5 [2, 3]	0.392
Normalized ADC*	0.83 [0.72, 0.95]	1.23 [0.92, 1.49]	**< 0.001**
Surgery type	Resection: 157 (68.0%) Biopsy: 74 (32.0%)	Resection: 6 (25.0%) Biopsy: 18 (75.0%)	**< 0.001**

*Median and interquartile range

Multiple logistic regression analysis revealed significant information about GBM type explained by the regression model (Wald X^2^ = 39.59, *p* < 0.001), with a model C-statistic of 0.91 (95% CI: [0.83, 0.99]). Adjusted odds ratios (AOR) for quantifying the association between GBM type and the MRI metrics and surgical type are displayed in Table 2. Contrast enhancement was positively associated with histological-GBM (AOR 7.83; 95% CI: [1.23, 49.68], *p* = 0.029). Ring enhancement was positively associated with histological-GBM (AOR 5.98; 95% CI: [1.09, 32.93], *p* = 0.040). Normalized ADC was negatively associated with histological-GBM (AOR 0.78; 95% CI [0.62, 0.99], *p* = 0.039). Vasogenic edema, hemorrhage, and surgery type were not uniquely associated with GBM type in the multiple logistic regression setting. Multicollinearity among the multiple regression predictors was assessed via the variance inflation factor (VIF) and the VIFs are presented in Table 3. Notably, all VIFs were well within the moderate (generally acceptable) multicollinearity range [1 < VIF < 5] implying the regression model specification for the systematic component (i.e., predictor variables) does not warrant variable scale/composition multicollinearity remedial measures.

**Table 2:** Multivariate exact logistic regression adjusted odds ratios for quantifying the association between the MRI metrics/surgical status and GBM type in the training cohort (0 = Molecular-GBM, 1 = Histological-GBM)

Predictor variable	Ratio	Odd Ratio [95% CI]	*P*-value
Contrast enhancement	Present: Absent	7.83 [1.23, 49.68]	**0.029**
Ring enhancement	Present: Absent	5.98 [1.09, 32.93]	**0.040**
Vasogenic edema	Present: Absent	3.32 [0.33, 33.69]	0.310
Multifocal	Present: Absent	1.63 [0.22, 12.30]	0.635
Maximum tumor diameter	X + 1:X	0.93 [0.67, 1.30]	0.678
Hemorrhage	Present: Absent	2.90 [0.62, 13.42]	0.174
Number of lobes involved	X + 1:X	0.50 [0.24, 1.05]	0.066
Normalized ADC	X + 0.1:X	0.78 [0.62, 0.99]	**0.039**
Surgery type	Resection: Biopsy	2.36 [0.55, 10.22]	0.250

**Table 3:** Multicollinearity variance inflation factor (VIF) for the training-set multiple logistic regression model, where VIF is a measure how much the variance of a regression coefficient is increased due to multicollinearity (correlation) among the predictor variables of the multiple regression model

Predictor variable	Multicollinearity VIF
Contrast Enhancement	1.56
Ring Enhancement	1.53
Vasogenic edema	1.17
Multifocal	1.74
Diameter of Whole Lesion	1.76
Hemorrhage	1.47
Number of Lobes Involved	2.08
Normalized ADC	1.23
Surgery Type	1.30

Note if VIF = 1 it indicates no multicollinearity (ideal), VIF < 5 it indicates moderate multicollinearity (generally acceptable), if VIF > 5 it indicates high multicollinearity (warrants model specification investigation) and if VIF > 10 it indicates serious multicollinearity (requires model specification action)

As the final step in the training dataset analysis, a reduced multiple logistic regression model was constructed to predict GBM type utilizing only the predictor variables in Table 2 that were uniquely associated with GBM type (contrast enhancement, ring enhancement, and normalized ADC). ROC analysis established an optimum diagnostic classification probability threshold of 0.85, where a predicted probability of ≥ 0.85 was classified as histological-GBM, and a predicted probability of < 0.85 was classified as molecular-GBM. When this classification rule was applied to the 231 histological-GBM training dataset patients, 222 patients (96.1%) were correctly classified as histological-GBM. When this classification rule was applied to the 24 molecular-GBM training dataset patients, 18 patients (75.0%) were correctly classified as molecular-GBM.

### Validation dataset analysis

Among the 44 GBM cases in the validation dataset, there were 32 histological-GBMs and 12 molecular-GBMs. There were 16 females (36.4%) and 28 males (64.6%). Median age was 63.0 years (interquartile range [IQR]: 52.5–71.0). There was no significant difference in age or gender between molecular-GBM and histological-GBM.

For the validation cohort, contrast enhancement was present in 32/32 histological-GBMs and 0/12 molecular-GBMs. Ring enhancement was present in 29/32 histological-GBMs and 0/12 molecular-GBMs. Mean normalized ADC for the histological-GBMs was 0.95 [95%CI: 0.87–1.02] and mean normalized ADC for the molecular-GBMs was 1.41 [95%CI: 1.12–1.71]. Predicted probabilities for histological-GBM were obtained for the validation dataset cases by inserting the validation dataset information for contrast enhancement, ring enhancement, and normalized ADC into the training dataset derived reduced multiple logistic regression model equation and converting the resulting predicted log-odds values (i.e., ln(θ)) to the probability (p) scale (i.e., p = e^ln(θ)^/1 + e^ln(θ^). Validation dataset patients whose predicted probability was ≥ 0.85 were classified as histological-GBM and validation dataset patients whose predicted probability was < 0.85 were classified as molecular-GBM. Application of the aforementioned diagnostic classification rule resulted in correct classification of all 32 histological-GBMs and all 12 molecular-GBMs in the validation dataset. Supplemental Figures S1 and S2 graphically depict the distributions of patient-specific predicted probabilities generated for the training-set reduced multiple logistic regression model and for the validation-set, respectively.

## Discussion

MRI features of glioblastoma were established on the basis of a histologically-based diagnostic standard [16], and included contrast enhancement, ring enhancement, low ADC values, increased cerebral blood volume, and elevated choline levels, among other metrics [17–20]. However, it was long known that some so-called “lower grade” gliomas behaved in an aggressive fashion, similar to GBMs [21]– [22]. The current WHO 2021 classification scheme now recognizes that the diagnosis of glioblastoma can be established in an IDH-wt diffuse astrocytic neoplasm either on basis of histologic features (microvascular proliferation and/or necrosis) or molecular features (TERTp mutation, EGFR amplification, or chromosome + 7/-10) [3]. The current diagnostic category of GBM is different than in the past, when nearly all radiologic studies were performed establishing the MRI appearance of glioblastoma. The current diagnostic category of Glioblastoma, IDH-wild type, CNS-WHO grade 4 contains many tumors that would have been previously classified as WHO grade II or III gliomas due to lack of microvascular proliferation and necrosis. It also no longer includes IDH-mutant gliomas that have microvascular proliferation and/or necrosis, namely most tumors in the current category “Astrocytoma, IDH-mutant, CNS-WHO grade 4” [3].

Our study establishes that, indeed, the majority of GBMs (which continue to be diagnosed on the basis of histologic features alone) demonstrate typical MRI features long associated with glioblastoma, including contrast enhancement, ring enhancement, edema, hemorrhage, and low ADC values relative to normal-appearing white matter. However, there is a significant minority of glioblastomas, namely those diagnosed on the basis of molecular features, that show distinct MRI features not typically associated with GBM. Such tumors often show no contrast enhancement, and have less frequent edema, less frequent hemorrhage, and higher ADC values compared to histologically-diagnosed GBM (Table 1). These results should be instructive to neuroradiologists and other members of neuro-oncology teams in the diagnostic work up of glioma patients. In particular, they support the point, with modern neuropathologic and neuroimaging data, that non-enhancing tumors on MRI should not be reflexively equated with “low grade gliomas” [23], and may warrant more prompt and aggressive management. It may be fruitful in future work to investigate MRI features that distinguish non-enhancing GBM from non-enhancing IDH-mutant gliomas, since many IDH-mutant gliomas lack contrast-enhancement on presenting MRI [24].

Our findings support the results of the few relevant studies in the literature. Guo et al. [6] investigated a cohort of 191 GBMs, including 146 histological-GBMs and 45 molecular-GBMs. They found that molecular-GBMs (vs. histological GBMs) were less likely to have contrast-enhancement (78.8% vs. 95.3%, *p* = 0.006) and intratumoral necrosis (63.6% vs. 85.3%, *p* = 0.005). Foltyn-Dumitru et al. [7] investigated 352 GBM IDH-wt and found that non-contrast-enhancing GBM (vs. contrast-enhancing GBM) less frequently had microvascular proliferation (39% vs. 94%) and necrosis (25% vs. 92%) (*P* < 0.001) on pathologic assessment, and were more likely to require molecular criteria for diagnosis (*P* < 0.001). Interesting, Lee et al. found that molecular-GBM has higher rates of gliomatosis growth pattern [8], but our results showed no significant difference in number of involved lobes, size, or multifocality between molecular and histological-GBM. The relative strengths of our study include inclusion of previously unreported MRI metrics and the use of multiple logistic regression statistical analysis to determine unique predictors of GBM-type. Furthermore, we validated our results using an independent cohort analyzed by a different neuroradiologist. Unlike prior studies, our study incorporated surgical status in our multivariate analysis. As expected, among glioblastomas in our training cohort, the requirement for molecular testing was more common when a surgical biopsy was performed (Table 1). This could reflect the fact that, with a surgical biopsy, there is greater risk of under-sampling and “missing” a tumoral sample which has necrosis or microvascular proliferation. It might also reflect the fact that diffuse non-enhancing glioblastomas are less optimal surgical targets for therapeutic resection and are thus more likely to be biopsied for diagnostic purposes only [18]. However, despite the strong association between surgical status and glioblastoma type in the univariate analysis, only MRI metrics remained significant predictors of glioblastoma type in our multivariate analysis.

It remains unclear whether and to what extent molecular and histological GBM are distinct biological entities and to what extent sampling bias play a role in the diagnostic pathway required for diagnosis. However, in addition to their distinctive MRI appearances, early evidence does appear to point to some clinical and biological differences between these GBM types. Patients with molecular-GBM appear to be slightly younger compared to patients with histological-GBM [6, 8]. Seizure is a more common presenting symptom for molecular-GBM than histological-GBM [6–8]. With regards to pathological findings, the evidence suggests that that molecular-GBM harbor higher rates of TERT-promoter mutation [8], while histological-GBM more likely harbor mutations of PTEN, TOP3A, CDK4, MYB, KIT, KRAS, and NTRK3 [6]. Ki-67 levels are observed to be lower in molecular-GBMs/non-contrast-enhancing GBMs [6, 7]. With respect to prognosis, Lee et al. found higher overall survival for molecular-GBM and Foltyn et al. found higher overall survival for non-contrast-enhancing GBM (comprised mostly of molecular-GBM), despite the fact that both these groups more likely underwent surgical biopsy than subtotal/gross total resection [7, 8]. Guo et al. found a non-significant trend towards longer survival for molecular-GBM [6]. If indeed molecular and histological-GBM prove to be distinctive biological entities, MRI features as established in our study and others may aid in accurate classification by helping to distinguish cases of true molecular-GBM (typically showing absent contrast/ring enhancement and higher ADC) from instances of under-sampled histological-GBM.

Our study has several limitations. We used a retrospective study design that would benefit from prospective validation. Our total number of molecular GBMs is low compared to histological GBMs resulting in a class imbalance common to other reports on this topic [6–8], and likely contributing to the large confidence intervals seen with our predictor variables in the multivariate analysis. Further the inclusion of contrast-enhancement and ring enhancement creates the potential for multicollinearity. However, the VIFs calculated for the predictor variables ranges from 1 to 2.25, indicating slight to moderate multicollinearity. Nonetheless, we acknowledge that future study would benefit from investigating GBM cohorts with a higher number of molecular GBMs and a narrower range of proven predictor variables, perhaps informed by the results of our study and other comparable studies. Our validation cohort was limited by its overall size (*n* = 44). Furthermore, our finding that the training set-derived prediction model performed better when applied to the validation cohort compared to the training cohort suggests that the validation cohort was in some way non-representative of the training cohort, or lacked the borderline cases necessary to rigorously test the model’s generalizability. We thus advise caution in the general application of our specific prediction model without additional independent validation. Differences in pathologic reporting between the training and validation institutions, including differences in sensitivity for microvascular proliferation, could have contributed to better performance of the model in the validation cohort, as this feature is known to correlate with increased probability of enhancement. While our choice of relatively simply MRI metrics helps ensure straightforward clinical application, it is also possible that advanced MRI techniques (including MR perfusion and MR spectroscopy) and AI-based classification tools could provide valuable insights into glioblastoma phenotypes. While minimum standard MRI pulse sequences were required for study inclusion, we did not perform all MRIs on the same scanner with uniform imaging parameters. Finally, we did not distinguish between molecular-GBMs on the basis of histologic features or patient age, factors that have been recently proposed for future GBM classification [25].

## Conclusion

Our study establishes that molecular-GBMs associate with absent contrast enhancement/ring-enhancement on MRI, and have higher ADC values compared to histological-GBMs. These associations are significant independent of surgical status. Recognition of the phenotypic variability of GBMs is important for neuro-oncology teams in the accurate pre-operative characterization of these lesions.

## Electronic supplementary material

Below is the link to the electronic supplementary material.

Supplementary Material 1

## Data Availability

The authors confirm that the data supporting the findings of this study are available within the article and its supplementary materials. If there are requests for additional data, they can be sent to the corresponding author (S.H.P.).

## Code availability (software application or custom code)

N/A.
