# Supplementary material for: Magnetic resonance imaging features differentiate histologic and molecular subtypes of glioblastoma IDH-Wild type CNS WHO grade 4
Source: J Neurooncol. 2026 Jan 21;176(3):177. doi: 10.1007/s11060-026-05431-8 (PMC12823664; doi:10.1007/s11060-026-05431-8)
Supplement: Supplementary file 1 — Supplementary Material 1 [file 11060_2026_5431_MOESM1_ESM.docx]

**SUPPLEMENTAL MATERIAL**

| Sequence | T1 MPRAGE | DTI | T2 SPACE | SWI | FLAIR |
| --- | --- | --- | --- | --- | --- |
| Plane | Axial | Axial | Axial | Axial | Axial |
| Mode | 3D | 2D | 3D | 3D | 2D |
| TR (ms) | 2300 | 5000 | 3200 | 27 | 8000 |
| TE (ms) | 3.25 | 95 | 412 | 20 | 83 |
| TI (ms) | 900 |  |  |  | 2372 |
| FA | 8° |  | T2 var | 15° | 150° |
| FOV read | 250mm | 220mm | 255mm | 230mm | 220 |
| FOV phase | 81.3% | 100% | 78.1% | 78.1% | 96.9% |
| NEX | 1 |  | 1.4 | 1 | 1 |
| Slice thickness | 1mm | 4mm | 1mm | 2mm | 3mm |
| Spacing | 0mm | 0mm | 0mm | 0mm | 0mm |
| Other | Acquired pre and post contrast | 20 directions |  |  |  |

**Table S1**. MRI protocol.

**Figure S1**. Predicted probabilities (p) for being of histological-GBM type among the training set of patients based the reduced training set multiple logistic regression model. Note the horizontal green hatch line at Y=0.85 represents the training set derived Youden optimum classification threshold, where patients of the training set who have a p <0.85 are classified as molecular-GBM, and patients of the training set who have a p≥0.85 are classified as histological-GBM. Based on the 4 quadrants defined by the intersection of green hatched horizontal line and black vertical solid line, true positive classifications are those histological-GBM patients correctly classified as histological-GBM patients, False negative classifications are those histological-GBM patients incorrectly classified as molecular-GBM patients, False positive classifications are those molecule-GBM patients incorrectly classified as histological-GBM patients, and True negative classification are those molecular patient correctly classified as molecular-GBM.

**Figure S2.** Validation predicted probabilities (VPP) for being of histological-GBM type among the validation set of patients based the multiple logistic regression coefficients derived from the training-set reduced regression model. Note the horizontal green hatch line at Y=0.85 represents the training set derived Youden optimum classification threshold, where patients who have a VPP <0.85 are classified as molecular-GBM, and patients who have a VPP ≥ 0.85 are classified as histological-GBM. Based on the 4 quadrants defined by the intersection of green hatched horizontal line and black vertical solid line, true positive classifications are those histological-GBM patients correctly classified as histological-GBM patients, False negative classifications are those histological-GBM patients incorrectly classified as molecular-GBM patients, False positive classifications are those molecule-GBM patients incorrectly classified as histological-GBM patients, and True negative classification are those molecular patient correctly classified as molecular-GBM.
